# Supplementary material for: Structural basis for loading of Transcription Repair-Coupling factor Mfd onto stalled elongation complexes
Source: bioRxiv. 2025 Sep 6:2025.09.05.674597. Preprint. [Version 1] doi: 10.1101/2025.09.05.674597 (PMC12424862; doi:10.1101/2025.09.05.674597)
Supplement: Supplement 1 [file media-1.pdf]

Supplemental Data

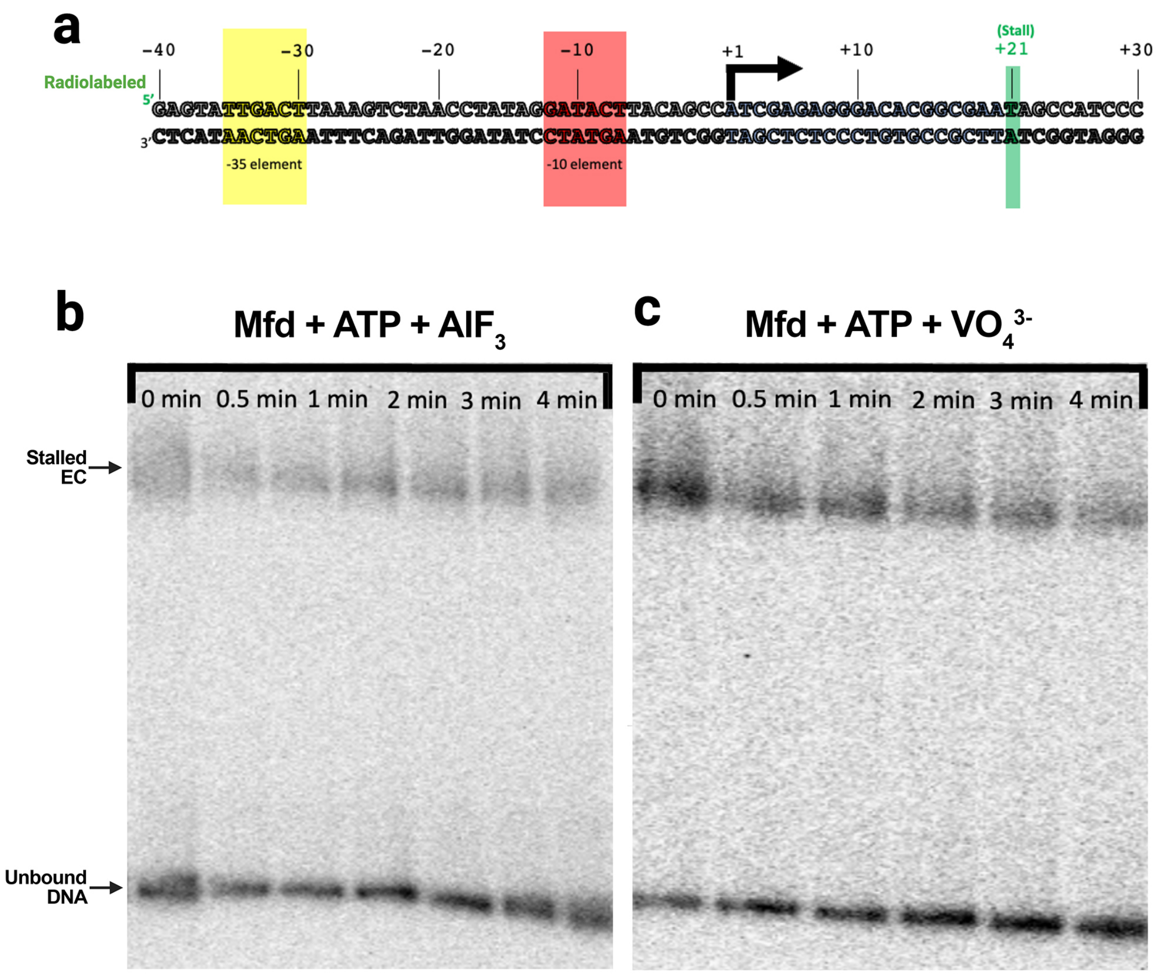

Supplementary Figure 1.

(a) Linear dsDNA T7A1 promoter fragment sequence used in native gel-shift experiments. Annotations include the core promoter elements (-35 element, yellow; -10 element, red), the transcription start site (+1, denoted with the bent arrow pointing in the direction of transcription), and the site of the EC stall (+21 shaded green, where RNAP is expected to incorporate chain-terminating 3'-deoxy-UTP). Position of g-^{32}P-ATP-labelled 5' end of the nt-strand is also shown in green.

(b, c) Representative native gel-shift results monitoring Mfd-mediated removal of stalled ECs in the presence of **(b)** AlF_3 (14 mM; left panel) or **(c)** VO_4^{3-} (20 mM; right panel).

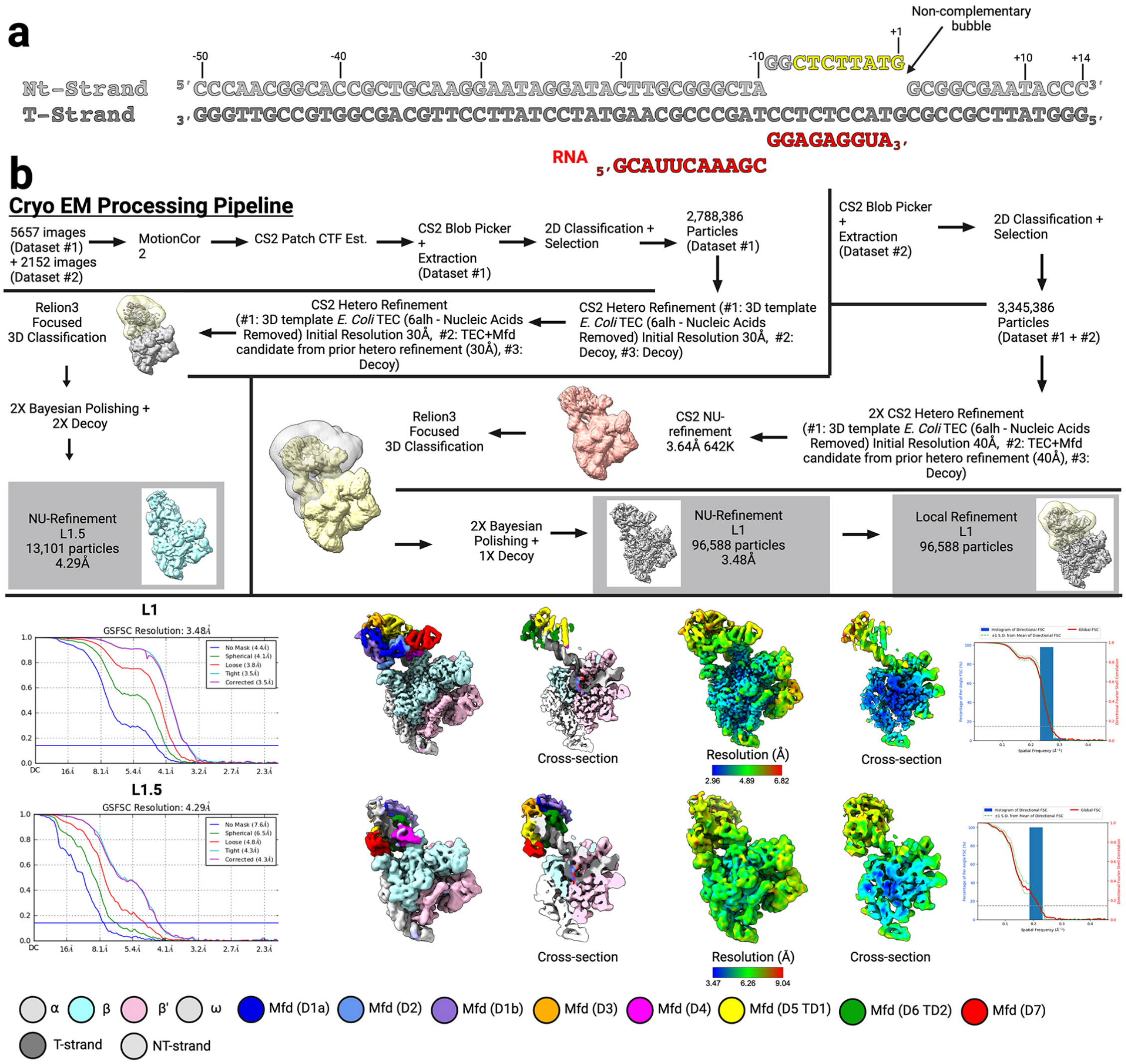

Supplementary Figure 2.

(a) Cryo-EM scaffold sequence highlighting partial complementarity between the RNA and t-strand DNA and between the t-strand and nt-strand.

(b) (Top half) Cryo-EM processing pipeline for L1 and L1.5 intermediates. (Bottom-half) Gold standard FSC calculations for cryo-EM density map [on left], cryo-EM density map and cross section colored according to key [middle-left], cryo-EM density map colored according to local resolution [middle-right] (Cardone et al. 2013), 3DFSC and sphericity of density map (Tan et al. 2017). L1 information shown in the upper row, L1.5 information shown in the lower row.

Supplementary Table 1 | Cryo-EM data collection, refinement and validation statistics for Mfd-EC. Related to Figures 2&3.

**Dataset**		
**Data collection and processing**	Dataset #1	Dataset #2
Microscope	FEI Titan Krios	FEI Titan Krios
Voltage (kV)	300	300
Detector	Gatan K3	Gatan K3
Electron exposure (e–/Å2)	51.2	68.2
Defocus range (μm)	1 to 2.5	1 to 2.5
Data collection mode	Counting Mode	Counting Mode
Pixel size (Å)	1.0825	1.0825 (binned from 1.03)
Symmetry imposed	C1	C1
Initial particle images (no.)	2,788,386	212,950
**Refinement**		
Structure	L1	L1.5
EMDB	EMD-48776	EMD-48802
PDB	9N07	9N11
Final particle images (no.)	96,588	13,808
Map resolution (Å) - FSC threshold 0.143	3.5	4.3
Map resolution range (Å)	3.0- 6.8	3.5- 9.0
Initial model used (PDB code)	6X26	6X50
Map sharpening B factor (Å2)	94.2	71.2
Model composition		
Non-hydrogen atoms	71,263	70,701
Protein residues	4,316	4,261
Nucleic acid residues (DNA/RNA)	113	113
Ligands	2 Zn^{2+}, 1 Mg^{2+}, 1 ADP, 1 BeF_3^-	2 Zn^{2+}, 1 Mg^{2+}
B factors (Å2)		
Protein	151.83	406.22
Nucleic acid	201.85	428.6
Ligands	283.02	279.08
R.m.s. deviations		
Bond lengths (Å)	0.005	0.004
Bond angles (°)	0.606	0.635
Validation		
MolProbity score	1.76	1.64
Clashscore	4.87	4.07
Poor rotamers (%)	0.17	1.0

Ramachandran plot		
Favored (%)	91.52	93.05
Allowed (%)	8.48	6.85
Disallowed (%)	0.0	0.09
